# Coherent phase transfer for real-world twin-field quantum key distribution

**DOI:** 10.1038/s41467-021-27808-1

**Published:** 2022-01-10

**Authors:** Cecilia Clivati, Alice Meda, Simone Donadello, Salvatore Virzì, Marco Genovese, Filippo Levi, Alberto Mura, Mirko Pittaluga, Zhiliang Yuan, Andrew J. Shields, Marco Lucamarini, Ivo Pietro Degiovanni, Davide Calonico

**Affiliations:** 1 INRIM, strada delle cacce 91, 10135 Torino, Italy; 2 INFN, sezione di Torino, via P. Giuria 1, 10125 Torino, Italy; 3 Toshiba Europe Ltd, 208 Science Park, Milton Rd, CB40GZ Cambridge, UK; 4 School of Electronic and Electrical Engineering, University of Leeds, LS29JT Leeds, UK; 5 Beijing Academy of Quantum Information Sciences, Building 3, West Area, No. 10 Xi-bei-wang East Road, Haidian District, 100193 Beijing, China; 6 Department of Physics and York Centre for Quantum Technologies, University of York, YO105DD York, UK

**Keywords:** Single photons and quantum effects, Quantum information, Fibre optics and optical communications, Optoelectronic devices and components, Optical metrology

## Abstract

Quantum mechanics allows distribution of intrinsically secure encryption keys by optical means. Twin-field quantum key distribution is one of the most promising techniques for its implementation on long-distance fiber networks, but requires stabilizing the optical length of the communication channels between parties. In proof-of-principle experiments based on spooled fibers, this was achieved by interleaving the quantum communication with periodical stabilization frames. In this approach, longer duty cycles for the key streaming come at the cost of a looser control of channel length, and a successful key-transfer using this technique in real world remains a significant challenge. Using interferometry techniques derived from frequency metrology, we develop a solution for the simultaneous key streaming and channel length control, and demonstrate it on a 206 km field-deployed fiber with 65 dB loss. Our technique reduces the quantum-bit-error-rate contributed by channel length variations to <1%, representing an effective solution for real-world quantum communications.

## Abstract (web summary)

Exploiting technologies derived from the optical clocks community, the authors demonstrate a setup for twin-field QKD which extends the coherence times by three orders of magnitude, overcoming the main challenge towards real-world implementation.

## Introduction

Quantum key distribution (QKD) enables to share cryptographic keys between distant parties, whose intrinsic security is guaranteed by the laws of quantum mechanics^1–4^. Besides pioneering experiments involving satellite transmission^5,6^, the challenge is now to integrate this technology on the long-distance fiber networks already used for telecommunications^7–16^. The maximum secure key rate for QKD decreases exponentially with the channel losses, an upper limit known as PLOB bound^17^, and although the reach could be extended using quantum repeaters, the related research is still at a rudimentary level, with far from operational devices^18–20^. Nowadays, intercity distances could only be covered using trusted nodes^14^, whose security represents however a significant technical issue.

**Fig. 1:**
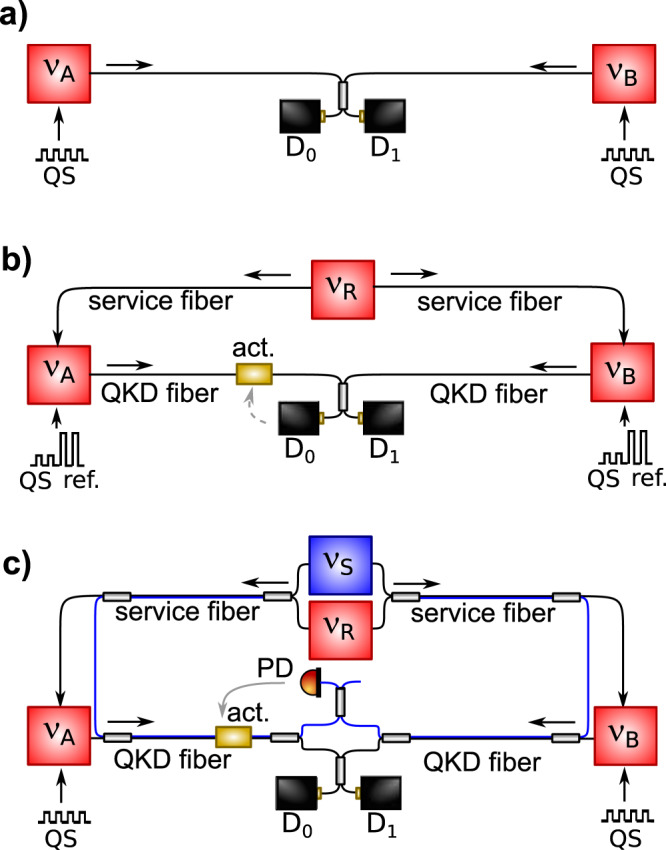
Principle schemes of TF-QKD. **a** In ideal TF-QKD, Alice and Bob encode quantum states (QS) on local lasers, attenuated to the single-photon level and with equal frequencies *ν*_A_ = *ν*_B_. The resulting signals are sent to Charlie, where they interfere on single-photon detectors (D_0_ and D_1_). **b** In practical implementations, a reference laser with frequency *ν*_R_ is sent to Alice and Bob through a service fiber, to phase-lock the QKD lasers and ensure *ν*_A_ = *ν*_B_ = *ν*_R_. After information encoding, QKD lasers are sent to Charlie through the QKD fibers. The transmission of QS is periodically interrupted to send reference phase states encoded in higher-intensity photon pulses (ref.), that allow detection of changes in the propagation path induced by length and refractive index fluctuations of the fiber. These are counteracted by either adjusting the phase of the incoming lasers through an actuator (act.) or by taking into account the instantaneous phase misalignment between the QKD fibers in post-processing. **c** In our approach, an additional sensing laser with frequency *ν*_S_ travels the service fiber with the reference laser, and the QKD fibers together with the QKD lasers. It can be spectrally separated because *ν*_S_ falls in a different channel of the dense wavelength-division multiplexed (DWDM) grid. While QKD lasers interfere on D_0_ and D_1_, the classical signals at *ν*_S_ are phase-compared on a photodiode (PD) to detect the noise of both the service and QKD fibers. This allows tight control of the fiber noise and simultaneous key streaming.

A fundamental resource for next-generation long-distance secure communications is represented by the recently proposed twin-field QKD (TF-QKD) protocol^21^, because of its weaker dependence on channel loss. In TF-QKD, the information is encoded as discrete phase states on dim laser pulses generated at distant Alice and Bob terminals and sent through optical fiber to a central node, Charlie, where they interfere. This idea, sketched in Fig. 1a, was proved secure against general attacks^22–26^ also in the finite-size scenario^27–29^ and with the aid of two-way communication^30^, but it is based on the critical assumptions that the optical pulses are phase-coherent in Alice and Bob and preserve coherence throughout the path to Charlie. While the first requirement can be fulfilled by phase-locking the two QKD lasers in Alice and Bob to a common reference laser transmitted through a service channel, the uncorrelated fluctuations of the length and refractive index of the fibers caused by environmental acoustic noise and temperature changes introduce phase noise to the system and reduce the visibility of the interference measurement. In proof-of-principle experiments based on spooled fibers^31–35^, this effect is mitigated by interleaving the QKD with classical transmission that provides information on the environmentally-induced noise and enables to periodically realign the phases of interfering pulses^31,32^ (see Fig. 1b). However, this approach becomes less effective as the length of connecting fiber spools exceeds few hundreds of kilometers^31,34,35^ and in deployed fibers^36^, where the attenuation and phase fluctuations are considerably higher and strongly dependent on the environmental conditions^37^.

We propose a solution derived from frequency metrology, where the transmission of coherent laser radiation over thousand-kilometer distances is employed to compare distant atomic clocks at the highest accuracy^38–44^ using ultrastable lasers and phase-stabilized optical fibers^45^.

In this work, we demonstrate that the same strategy can be exploited in TF-QKD and realize a setup where the phase fluctuations of both the lasers and connecting fibers are actively canceled. We implement our solution on a real-world network where the distance between Alice and Bob is 206 km and the net losses are as high as 65 dB, demonstrating a significant progress in the coherence time of the interfering signals as compared to previous implementations^31–36^. We adopt a wavelength-multiplexed approach in which an additional laser is sent in the same fiber as the QKD lasers and used in Charlie to sense and stabilize the channels’ optical length by interferometric means (see Fig. 1c). In a QKD experiment, this allows simultaneous key streaming and channels stabilization, ensuring more advantageous duty-cycles and a tighter control of the optical phase on long-haul deployed fibers, where interleaved approaches would fail. Our work establishes a clear connection between the optical clocks and quantum communication fields in terms of technologies and expertize, paving the way for a higher degree of integration towards long-distance quantum secure communications.

## Results

**Fig. 2:**
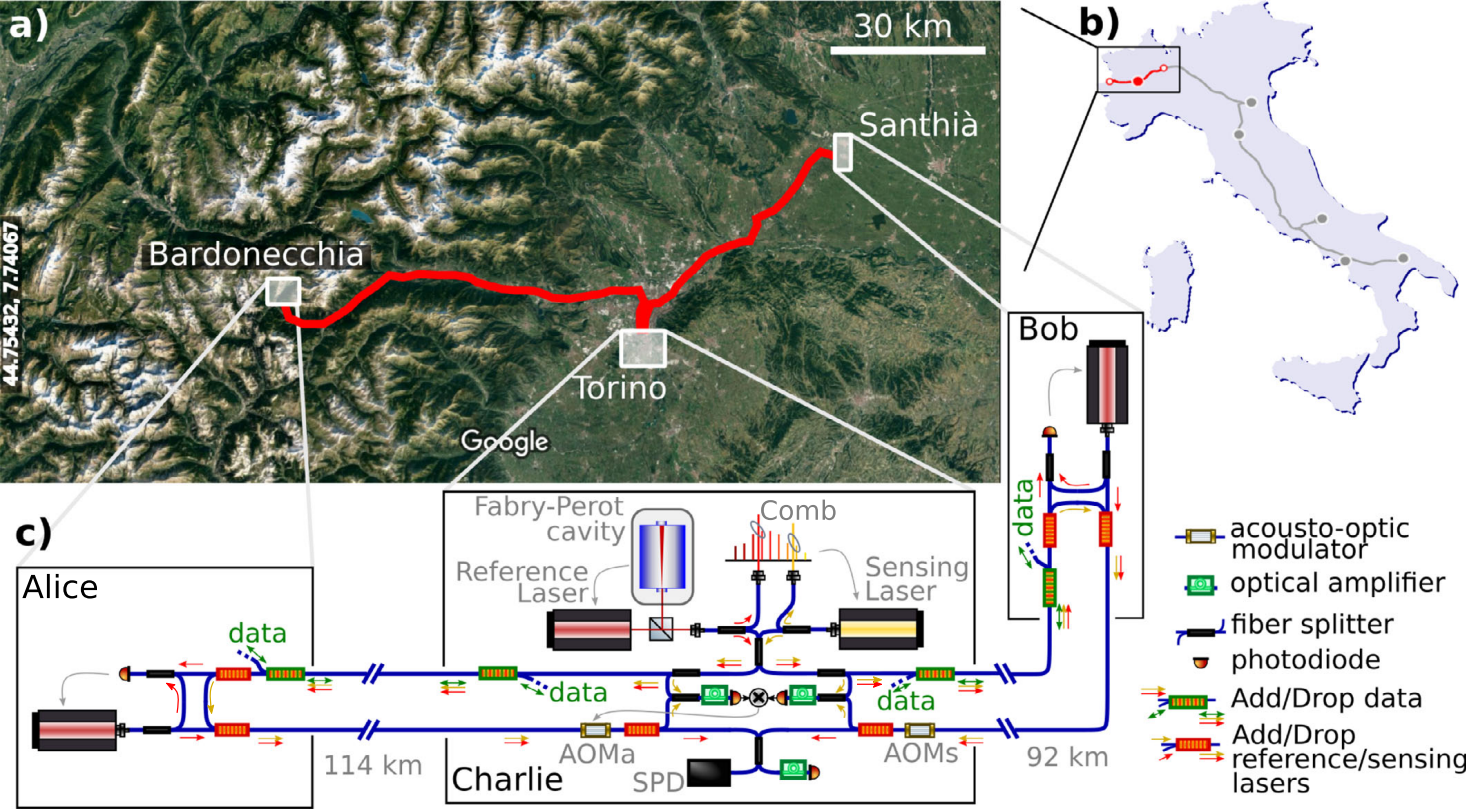
Map and experimental set-up. **a** The testbed layout, with the Charlie node located at INRIM (Torino) and the Alice and Bob nodes in shelters of the telecom network in Bardonecchia and Santhià (Imagery ^©^2020 Landsat/Copernicus, Imagery ^©^2020 TerraMetrics, Map data ^©^2020). Latitude and longitude are indicated on the left edge. **b** A sketch of the Italian Quantum Backbone, with the spans used in this experiment colored in red and the red-filled (empty) circles representing Charlie (Alice and Bob). **c** The experimental setup. The reference laser in Charlie is stabilized to a high-finesse cavity and the sensing laser is phase-locked to it using an optical comb. They are combined and injected in the service fibers (the uppermost ones) together with bidirectional data signals using wavelength division multiplexers. In Alice and Bob, the reference and sensing lasers are separated using the same devices. We detect the beat between the incoming reference laser and the local QKD lasers and phase-lock the two. The QKD lasers are then recombined with the sensing laser and sent to Charlie on dedicated fibers (the lowermost ones). Upon recombination, the QKD lasers interfere on a photodiode when performing experiments in the classical regime, and on a single photon detector (SPD) in the photon-counting regime, also used to detect background photons. The fiber noise is detected by interfering the local sensing laser with return light from each arm. The two beatnotes are detected on separate photodiodes and phase-compared in the RF domain. The resulting signal is integrated in a proportional-integral analog feedback loop and applied to the driving frequency of the acousto-optic modulator AOMa, which in turns adjusts the optical phase of the travelling beams to stabilize the interferometer. Because both the sensing and QKD lasers travel through AOMa, the fiber noise is suppressed from the QKD interference signal as well. AOMs is a fixed frequency shifter, used to enable the self-heterodyne beatnote detection on Bob’s branch.

The map and detailed scheme of our experiment are shown in Fig. 2. We use a pair of ultrastable lasers with a linewidth of ~1 Hz and frequency *ν*_R_ = 194.4 THz (1542.14 nm) and *ν*_S_ = 194.25 THz (1543.33 nm), which are standard frequencies of the dense wavelength-division multiplexed (DWDM) grid. The former (hereafter, reference laser) is used as a reference for locking the QKD lasers in Alice and Bob terminals, and is frequency stabilized to a high-finesse ultrastable Fabry−Perot cavity^46^. The latter (hereafter, sensing laser) is used to detect the fiber noise and allows its cancellation. In our experiment, we offset-locked it to the reference laser using an optical frequency comb^47^, although alternative techniques are possible (see “Methods”). Cavity-stabilized lasers and frequency combs, the most stable of which are used as local oscillators in optical clocks and tick synchronously with narrow atomic resonances, are standard devices in the frequency metrology community and are now highly-integrated and commercially available systems. The two ultrastable lasers are combined using a commercial 100 GHz-wide DWDM filter and sent to Alice and Bob through separate service fibers.

At the remote terminals, the reference laser is extracted and used to phase-lock the local QKD laser, which in turns is recombined with the sensing laser and sent back to Charlie on the QKD fiber. This setup includes what is needed for transmitting quantum information, nonetheless, we do not realise a fully-operative QKD transmission since this is a technical aspect of the implementation already demonstrated elsewhere^31–34^, very recently also in a real-world testbed^36^. Instead, our experiment focuses on improving the system coherence, which is the essential prerequisite for any TF-QKD protocol. In the Supplementary Information, we provide a detailed overview of the additional components required for implementing TF-QKD and their integration into the present scheme.

In Charlie, we interfered the QKD lasers in classical and photon-counting regimes, the latter after attenuating them to the single-photon level. As the sensing laser travels the path from Charlie to Alice and Bob together with the reference laser on the service fibers, and the backward path together with the QKD lasers on the QKD fibers, its accumulated phase contains information on the changes of propagation paths in the whole interferometer, that can be used to stabilize it. The incoming beams at the two wavelengths are routed to separate detectors: photons from the QKD lasers interfere on a photodiode (when doing experiments in the classical regime) or a single-photon detector (SPD, in the photon counting regime), while the sensing laser beam that traveled a round-trip in each arm is interfered on a photodiode with a portion of the local laser in a self-heterodyne configuration. The two resulting beatnotes are phase-compared on a radio-frequency mixer in quadrature condition that produces an analog signal proportional to the phase difference between the two interferometer arms. This signal feeds a proportional-integrative analog control loop that adjusts the phase of the travelling beam on Alice’s branch, by acting on the driving signal of the acousto-optic modulator AOMa. Because the QKD laser also travels through AOMa, the same correction is applied to it, effectively suppressing the fiber noise from the QKD interference signal (see “Methods” for more details).

We implemented this scheme over long-haul fiber backbones connecting INRIM, in the city of Torino (Italy), where the Charlie terminal was located, to network nodes separated by 114 and 92 km of optical fiber with 35 and 30 dB losses (Alice and Bob terminals respectively). The overall length of the fiber connecting Alice and Bob was thus 206 km with an attenuation as high as 65 dB. The average loss coefficient of 0.3 dB/km is higher than the specified level for standard optical fibers (0.2 dB/km) and includes discrete losses of the connectors and DWDM equipment, which play a significant role in deployed networks. These fibers are part of the Italian Quantum Backbone and carry other services, among which is the dissemination of atomic clock signals to research facilities of the Country^38,39^. Conventionally, telecom networks support two-way data exchange over fiber pairs, in which each fiber allows light propagation in a single direction. On our testbed, in collaboration with the fiber provider, we implemented instead a bidirectional transmission on single fiber, using different DWDM channels for opposite directions. Using this approach, the second fiber of the pair was dedicated to the sensing and QKD lasers only (see “Methods”), while the service fibers carry, in addition to the reference and sensing lasers, also standard data traffic from other network users and time/frequency dissemination services. Specifically, this latter can be conveniently exploited in a TF-QKD experiment to synchronize the clock signals at the remote terminals and implement ultra-precise timing of the quantum states encoding. Here, we used a White Rabbit precise time protocol^48,49^ to distribute clock information for the optical phase-lock of the QKD laser in Bob.

**Fig. 3:**
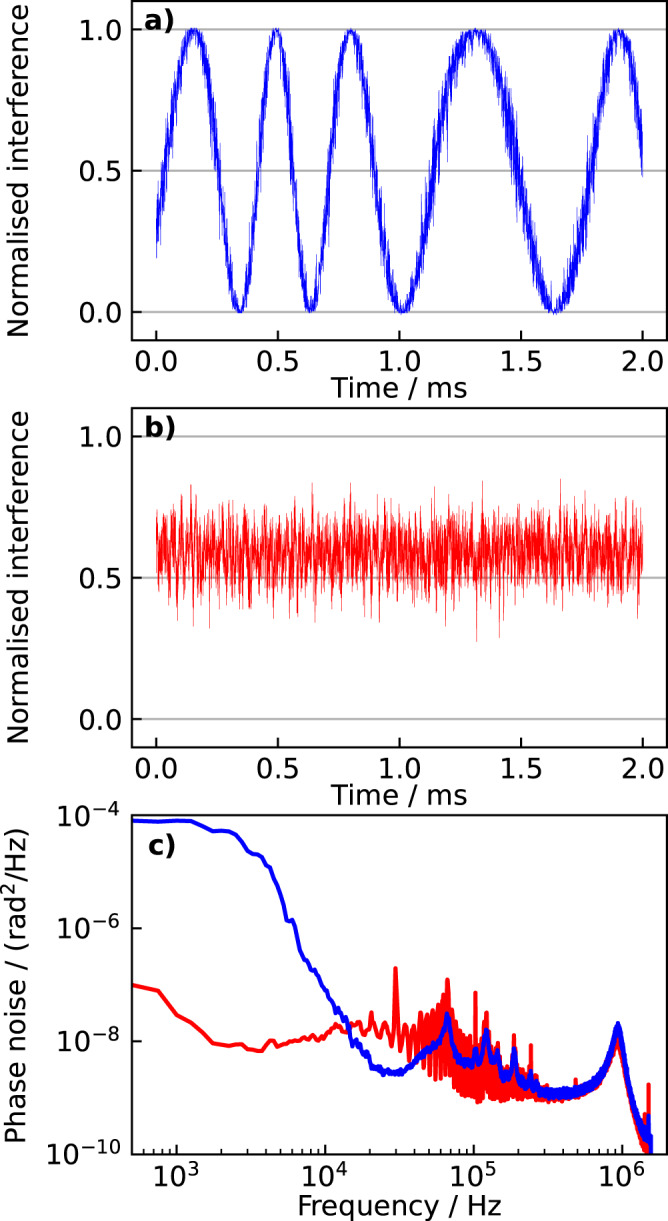
QKD lasers interference with unstabilized and stabilized fibers. We record the interference between the QKD lasers in Charlie on a fast photodiode (the traces are normalized between 0 and 1. **a** In an unstabilized condition the instantaneous phase drifts by 30 rad/ms and is folded back when it exceeds the [0: *π*] interval. **b** When the fiber is stabilized, the phase remains stable. In this measurement, the interferometer was stabilized far from the folding point, i.e., in a condition where phase fluctuations were directly mapped into intensity fluctuations, to investigate the residual noise processes. **c** The power spectral density of the phase. A significant reduction in the noise is observed in a stabilized condition (red) with respect to an unstabilized condition (blue). The apparent plateau observed at Fourier frequencies below 3 kHz in an unstabilized condition is an artifact originated by the folding of the interferometer response. At high Fourier frequency, similar noise is observed in the two traces, mainly due to the residual QKD lasers noise within the locking bandwidth, whose servo bumps are clearly observed around 900 kHz, and the self-delayed interference of the reference and sensing lasers, which give rise to the characteristic ripples pattern in the Fourier frequency range between 10 and 100 kHz (see Supplementary Information for a detailed analysis).

Figure 3 shows the interference between the QKD lasers measured on a photodiode in Charlie in a 2 ms time frame, without (a, blue) and with (b, red) active stabilization of the fiber paths. In an unstabilized condition, several phase cycles are accumulated in the considered interval, with an instantaneous drift of up to 30 rad/ms. When the path is stabilized, on the contrary, the phase remains stable over the whole acquisition frame. In this measurement, the phase was stabilized on purpose at a point where its fluctuations were directly mapped into intensity fluctuations, which enabled us to compute the corresponding phase noise power spectral density. This is shown in Fig. 3c: in an unstabilized condition (blue) the phase noise rapidly diverges at low Fourier frequencies while it is suppressed up to a bandwidth of tens of kilohertz when stabilization is activated (red). In both traces, the noise floor is set by the QKD lasers noise at and within the locking bandwidth of 0.9 MHz and the self-delayed interference of the reference and sensing lasers. These contributions are common to the two traces. The latter becomes proportionally higher as the length unbalance between the arms of the interferometer increases or the spectral purity of the lasers degrades^45^. The use of ultrastable lasers with 1 Hz linewidth was crucial in our setup, where the unbalance was 22 km, i.e., 44 km of differential path considering both the service and QKD fibers (see the Supplementary Information for a complete frequency-domain analysis). As the relevant noise processes extend up to ~1 MHz Fourier frequency, we note that an acquisition system with a minimum measurement bandwidth of 2 MHz is required to fully capture them. Devices with slower frequency response would act as low-pass filters, leading to underestimation of the corresponding phase changes.

**Fig. 4:**
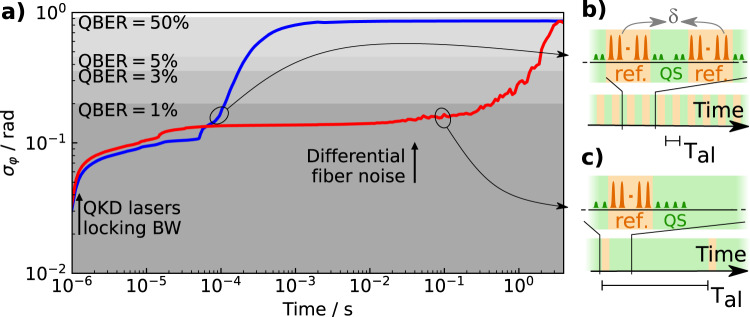
Phase fluctuations over time. **a** The deviation of the phase *σ*_*φ*_ between the two QKD lasers interfering in Charlie at different timescales, in an unstabilized (blue) and stabilized (red) condition. For this calculation, we acquired the interference pattern over 4 s and subdivided it into shorter time frames, calculating *σ*_*φ*_ for each frame. The shadowed areas indicate upper thresholds for relevant values of the QBER. The phase and corresponding QBER were retrieved from the interference pattern according to the procedures described in the “Methods”. The arrows indicate timescales where the QKD lasers noise and the uncompensated fiber noise (differential fluctuations at the two wavelengths) become relevant. For a given TF-QKD implementation, the quantum states transmission (QS) must be interleaved with realignment frames encoding a reference phase (ref.) after a time *T*_al_. This allows detection and stabilization of the interferometer noise *δ*, and ensures that a specified QBER is not exceeded: in our case, to preserve a QBER < 1%, *T*_al_ amounts to 100 μs and 0.1 s using an unstabilised or stabilized interferometer respectively. While in the former case realignment frames are absolutely required (**b**), in the latter case it becomes possible to exploit variations in the QBER itself to derive information about the interferometer phase noise and stabilise it. Even in the worst case scenario in which this is not possible because of a too high fiber loss, realignment frames need to be applied at a much slower rate (**c**), effectively enabling duty cycles higher than 90% (see Supplementary Information).

The QBER associated to phase-decoherence can be calculated from the standard deviation of the phase *σ*_*φ*_ as a function of the frame duration (see “Methods”). Figure 4 shows the results in a stabilized (red) and unstabilized condition (blue). In both cases, the noise processes responsible for phase fluctuations extinguish at timescales shorter than the inverse of the locking bandwidth of the QKD lasers (indicated by the arrow), making *σ*_*φ*_ negligible for integration times below 1 μs. However, in an unstabilized condition, the system exceeds the 1% QBER threshold in about 100 μs. At timescales longer than a few milliseconds, apparently, the phase fluctuations do not increase further. This is an artifact caused by the limited range of the interferometer response, which wraps the phase into the [0; *π*] interval. In practice, the phase wanders by tens of radians in few milliseconds. When stabilization is activated, instead, the QBER is kept below 1% for about 400 ms. Notably, for integration times of up to 100 ms the system remains in a condition where *σ*_*φ*_ = 0.13 rad, which corresponds to a QBER of 0.5%. This value is determined by the residual contribution of the reference, sensing, and QKD lasers noise. The increase in *σ*_*φ*_ which is observed for longer times is due to a non perfect cancellation of the fiber noise and depends on the fact that the measurement is based on the accumulated phase of the sensing laser, while the reference and QKD lasers accumulate a slightly different phase because of the wavelength difference and uncommon optical paths (see Supplementary Information). This effect is largely predictable and could be reduced with an optimized design of the experimental setup and dedicated electronics. Moreover, the residual phase drift can be detected using the same strategy as in previous TF-QKD implementations^36^, where periodical realignment frames are interleaved with the quantum transmission. Then, it can be canceled e.g., using an additional phase modulator or fiber stretcher on one of the quantum signal paths, after it is wavelength-separated from the sensing laser in Charlie. However, in our case, the periodicity for these realignment frames is reduced by three orders of magnitude (see Fig. 4b and c) as compared to other implementations.

The observed residual phase noise and QBER represent conservative estimates, as our measurements were performed on a testbed with as much as 22 km of unbalance between the two arms and with standard telecom diode lasers at the Alice and Bob terminals featuring 3 kHz Lorentzian linewidth and 0.9 MHz control bandwidth. Further improvement could be gained using less noisy telecom lasers^50,51^ and faster control techniques. However, already in the present condition, the system maintains a QBER < 3% for timescales of the order of 1 s.

**Fig. 5:**
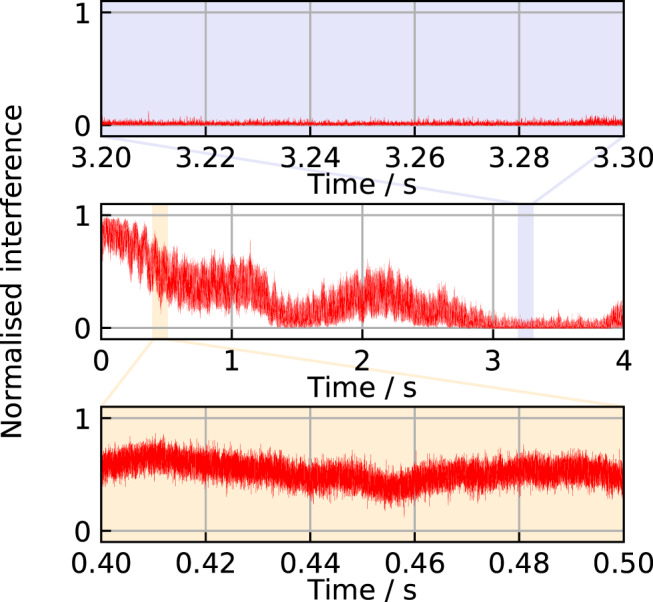
QKD lasers interference on the long term. In the central panel, the normalized interference pattern in the stabilized-fiber condition is shown in a 4 s-long time window. The shadowed area zoomed in the uppermost panel indicates a 100 ms-long fraction of the time interval in which the interferometer operated in the maximum visibility condition, i.e., the one exploited for the exchange of the bits of the cryptographic key. The shadowed area zoomed in the lowermost panel indicates a 100 ms-long time interval in which the interferometer operated around the 0.5 visibility. Configurations far from the deterministic behavior of the interferometer (i.e., far from the maximum and minimum of the visibility) are the ones where the relation between the phase fluctuations and the QBER is linear, and could be used to realign the phase on the long term and mitigate the residual uncontrolled optical path length variations (see Supplementary Information).

Figure 5 shows the interference pattern on a 4 s timescale, and a zoom of a 100 ms-long period where the system could be operated at the maximum visibility in a QKD experiment. We also show a zoom of a 100 ms-long region where the interferometer operates far from the deterministic condition. With such a stability, in a QKD experiment, it becomes possible to gather enough photon statistic for realigning the phase on the basis of the QBER (see Supplementary Information). This approach can be applied as long as the optical loss of the interferometer is not the limiting factor, thus releasing the need for periodical realignment frames and virtually ensuring uninterrupted operation.

The same measurements were repeated by attenuating the QKD lasers at the remote terminals by ~80 dB, so that only few thousands of photons/s reach the detector in Charlie, under similar operating conditions as in recent TF-QKD experiments^31–34^. For this measurement, we replaced the photodiode with an SPD and recorded the number of counts as a function of time. We were able to reproduce the same visibility as with the classical beams, showing substantial agreement between the two approaches. This aspect is treated in detail in the Supplementary Information.

In view of the implementation of this technique in a QKD experiment, an aspect of concern is the control of the background photons that couple to the QKD fibers from the surrounding environment, or are originated in the QKD fibers themselves due to nonlinear effects. Only photons at the QKD lasers wavelength are relevant to the count, as those in other bands can be filtered out. To counteract the drop in performances of standard DWDM filters outside the C-band, we combined them with broader filters featuring 50 dB attenuation throughout the visible and near infrared spectrum (see “Methods”). This ensures efficient separation of the sensing and QKD lasers photons, and provides adequate immunity to background photons from external sources even when the network occupancy and its spectral distribution are unknown.

Background photons in the same band as the quantum signal are mainly produced by the Raman scattering of the sensing laser in the QKD fiber. This problem is well known in the context of real-world QKD and forces to use either dedicated fibers or QKD transmission in the O-band at 1310 nm, where the scattering from channels in the C-band is negligible^10,52^. In our experiment, we could minimize its impact by ensuring that the sensing laser power coupled to the QKD fiber at the Alice and Bob terminals was <1 μW. Another effect contributing photons in the same band as QKD lasers is the Rayleigh scattering of the reference laser happening in the service fiber. Rayleigh-scattered photons propagating backward into the service fiber may fall into the QKD fiber due to evanescent coupling. This effect can be mitigated by reducing the launched power of the reference laser in the service fiber. In our case, the maximum allowed power was 20 μW, which was still enough for ensuring a stable phase lock of the slave lasers at Alice and Bob terminals. Because this effect is stronger in the first ~20 km from Charlie, optical amplifiers can be inserted close to the Alice and Bob terminals along the service fiber to cover longer distances or more lossy paths. These devices do not affect the phase stability, as extensively demonstrated in the context of metrological frequency transfer^53^. In this specific application, however, the same optical amplifier should be preferentially used for the reference and sensing beam, to avoid residual phase drifts due to non-common optical paths between the two.

We measured the background photon rate in our setup exploiting a low noise InGaAs/InP avalanche photodiode with quantum efficiency of 10% and dead time of 25 μs. In the working conditions the observed rate of background photons was (5.09 ± 0.01) s^−1^, evaluated over 24 h of measurement, primarily attributed to Raman scattering of the sensing laser. When all the laser sources involved in our experiment were switched off, the measured level of background photons was (4.76 ± 0.04) s^−1^, slightly above the intrinsic dark count rate of our SPD, i.e., (4.52 ± 0.03) s^−1^, meaning that the background photons flux coupled from nearby fibers or from the metropolitan environment is minimal. Overall, the background photon rate introduced by our apparatus is of the same order of the dark count rate of our SPD, and is expected not to significantly affect the QBER.

## Discussion

We realized a setup suitable for TF-QKD and characterized it over a 206 km-long deployed fiber with 65 dB of optical loss. The setup implementation in real-world conditions required addressing several fresh challenges, such as a considerably higher attenuation than on spooled fiber (0.3 dB/km on our setup), autonomous and remote-controlled operation of the equipment at the Alice and Bob terminals, which was deployed in telecom shelters in a non-controlled environment, and a considerable unbalance (22 km) in the interferometer arms. Even under these conditions, we ensured the phase coherence of interfering lasers over hundreds of milliseconds, i.e., 1000 times more than what reported so far in laboratory trials, which in turns ensures longer duty-cycles for the QKD protocol implementation. Furthermore, besides temperature, acoustic and seismic noise on the fibers, our scheme also compensates non-stationary events such as those due to human activities, and is expected to be robust against further up-scaling of the infrastructure in terms of length, attenuation, and phase noise.

The key points of our technique lie in the use of ultrastable lasers with several hundreds of kilometers of coherence-length, which is mandatory considering the constraints set by the network topology which prevents from realizing perfectly-balanced interferometers, and the simultaneous transmission of separate signals for the fiber noise detection and the key streaming. On one hand, this enables to keep the QBER to manageable levels thanks to a tighter control of the phase; on the other hand, it allows more advantageous duty cycles for the quantum communication. In our experiment we were able to maintain *σ*_*φ*_ = 0.13 rad, corresponding to a QBER of 0.5%, for about 100 ms. Both aspects concur to increase the effective key rate, which is a major advantage especially on long haul networks, where rather low rates of a few kb/s must be already taken into account due to the fiber losses^31^. We performed a detailed simulation to estimate the achievable key-rate in a TF-QKD protocol implementation with active phase-noise cancellation, considering the protocol proposed in^25^ in the asymptotic scenario with decoy states. The results show that owing to the low phase jitter enabled by active phase stabilization, a secret key rate of up to 4.942 kb/s can be achieved, with a gain of 10.83 over the PLOB bound. Note that in our simulation we conservatively set the QBER to 1.18%. With active noise cancellation, we are always below this threshold at integration times of 100 ms (see Fig. 4). On favorable conditions where the optical loss is not the limiting factor, monitoring the QBER can allow the long-term stabilization of the phase. However, even in the case this is not possible, alignment frames can be conducted every 100 ms. In both cases, the estimated duty-cycle is higher than 90%: with such an high value, our simulations in asymptotic regime are close to real. The complete set of parameters used for this simulation are reported in the Supplementary Information.

We note that the realized scheme allows rejection of the service fiber noise as well. While previous implementations focus on the strategies for mitigating the noise on the QKD fiber, the issue of noise on the service fiber was only marginally addressed so far^34,54^, proposing the Doppler noise cancellation^45^ as an effective solution. However, we note that this approach is bandwidth-limited by the time needed by the light to travel the fiber, and would leave several radians of uncompensated phase fluctuations in a realistic case^45^. In our multiplexed scheme, on the contrary, the noise detection is performed upon recombination in Charlie, and the correction can be applied without delay, thus ensuring a higher suppression. Another approach exploits a Sagnac-interferometer-based configuration^33^, which, however, suffers from the same limitation as the Doppler stabilization^55^. On the basis of the results obtained in this work, we also foresee the Rayleigh effect as a major source of background photons in a Sagnac loop. Rayleigh scattering poses a limitation on the maximum distance achievable when time division multiplexing is employed^34^, while the wavelength division multiplexing strategy we are proposing is free from this limitation. We also note that the results of this work are not related to any specific system architecture, and describe useful strategies for a variety of QKD protocols.

The proposed scheme can be directly implemented in real-world quantum communication systems. We underline that, together with phase fluctuation, there are other non-ideal behaviors in the encoding pattern (modulation and phase) of the QKD lasers at the remote terminals that may reduce the visibility of the interference and increase the QBER. Among these, are the relative jitter of the clocks referencing the patterns in Alice and Bob, and the pulses’ arrival time in Charlie which is in principle affected by the varying delay added by the fibers. In this perspective, we note that our proposed technique, being originally developed to cope with related issues in metrological applications, supports a stable and common clock signal to be delivered to the terminals through the service fiber. The additional timing delay jitter introduced by the QKD fibers is estimated to be <1 ps. Thus, provided that the modulation patterns are initially matched to account for the different lengths of the interferometer arms, it is not expected to affect the visibility of the interference even at the high modulation rate of ~1 GHz. However, the multiplexed approach used to stabilize the optical phase could be further exploited to compensate for the overall jitter of the service and QKD fibers.

Motivated by the increasing accuracy of state-of-the-art optical clocks, ultrastable frequency distribution over fiber is becoming a widespread technique and has found several practical applications, ranging from fundamental physics to distributed sensing and space-geodesy^38,56,57^. In this perspective, TF-QKD can be considered a further use-case for this technology. We demonstrate its use to stabilise a TF-QKD interferometer to a level where residual phase-fluctuations remain negligible up to unprecedented timescales. We foresee the convergence of physical layers, devices, and technologies between the quantum communication and atomic clock communities as an enabling tool within the new quantum revolution, that could open the possibility of advanced distributed networks integrating quantum sensors, optical clocks, and secure quantum communication protocols.

## Methods

### The optical fiber network

The fibers used for this experiment are part of the Italian quantum backbone, which provides atomic clock dissemination services to scientific and commercial users of the Country^38^. These services, as well as standard data traffic for the remote control of the equipment at the network nodes, require bidirectional optical transmission. To ensure compatibility with QKD, we migrated the traffic to the single service fiber, using different wavelengths for the two directions of propagation. In particular, time/frequency dissemination services used channel 30 and 31 of the International Telecommunication Union (ITU) grid, corresponding to a wavelength of 1553.33 and 1552.52 nm respectively. Remote control was established over channels 28 and 29 (wavelengths 1554.94 and 1554.13 nm). Standard DWDM multiplexers were used to combine and separate channels 28−31 at the network nodes, while the sensing and reference lasers traveled through the unfiltered ports, which brought ~2 dB additional losses each.

### Reference and sensing lasers

The reference laser is a fiber laser at 1542.14 nm (channel 44 of the 100 GHz-DWDM grid), frequency stabilized to an ultrastable Fabry-Perot cavity with a Finesse of 120,000 using the Pound−Drever−Hall technique^46^. The resulting linewidth is 1 Hz and the short-term instability is 2 × 10^−15^. The cavity is made of ultra-low expansion glass, housed in high vacuum, and placed on a platform for passive seismic noise damping. We used an external acousto-optic modulator (AOM) as a fast actuator to lock the fiber laser to the cavity. The achieved bandwidth of 200 kHz is limited by the internal delay of the AOM and by its driver. Although diode lasers offer much higher control bandwidths, their phase noise is higher as well, which deteriorates the phase coherence and makes the use of fiber laser preferable for this application. The reference laser is a diode laser at 1543.33 nm. This wavelength lies in the middle between the channels 42 and 43 of the 100 GHz-DWDM grid, and is a standard of the advanced 50 GHz-DWDM grid. We virtually phase-locked it to the reference laser using an optical comb as a spectral bridge. The comb is an octave-spanning Er:fiber femtosecond frequency comb with 250 MHz repetition rate whose spectral emission is centered around 1560 nm. Following the technique described in^47^, we detected the beatnote of both lasers with the comb and measured their phase-difference on a mixer. This enabled us to detect the relative phase between the two, which could not be directly measured because of the large spectral separation. The phase error was then used to phase-lock the sensing laser acting on its current. This approach is preferable than the use of independent optical cavities, because it ensures a tight phase-coherence between the reference and sensing lasers, which mitigates the impact of self-delayed lasers noise on the QKD interference pattern. This is possible because the self-delayed noise of the sensing laser is detected alongside with the optical fiber length variations and equally canceled by the stabilization loop. As long as this noise is common with the reference laser, it is rejected from the QKD interference as well. An opposite mechanism would take place using independent lasers, in which case the self-delayed noise of the sensing laser would be written onto the QKD interference. A tight phase relation between the two lasers could be maintained even without using a frequency comb, relying on fast electro-optic modulators and sideband-locking^56^.

### Phase lock of the slave diode lasers

About 20 μW of the reference laser power was launched in the service fiber towards Alice and Bob terminals. Here, commercial diode lasers with an optical power <10 mW were phase-locked to it. To this purpose, we detected the beatnote between incoming and local light on a fast photodiode and compared it to a stable radio-frequency oscillator on a mixer in a quadrature condition. The phase error signal was processed by a proportional-integrative-derivative controller acting on the laser current. The phase-locked-loop bandwidth of 0.9 MHz results from a combination of the frequency vs current response of the diode and the current driver’s bandwidth.

The local oscillator for the phase-locked loop in Bob (Santhià) was referenced to a 10 MHz signal disseminated with a White-Rabbit Precise Time Protocol over the service fiber, with short-term frequency stability of 1 × 10^−11^ ^48,49^. In Alice (Bardonecchia), where this service was not activated, we used a Rubidium oscillator with short term stability of 5 × 10^−12^.

### Cancellation of the fiber phase noise

The launched power of the sensing laser was about 1 mW into each arm of the interferometer. We stabilized the relative phase between the two return beams incoming in Charlie after travelling the path toward the remote terminals and back. To do so, we spilled out a portion of the sensing radiation before sending it to the remote terminals, and we detected the beatnote with the return signal on each arm on a photodiode. The resulting beatnotes at 40 MHz, the AOMs and AOMa frequencies, are down-scaled by a factor of 10 and phase-compared on a mixer in quadrature condition. The resulting error signal drives a proportional-integrative controller which adjusts the driving frequency of AOMa. As a result, the phase of the optical beams that pass through it, i.e., both the sensing and QKD laser, is dynamically corrected and compensates for the relative phase-fluctuations of the optical paths in the two arms. The use of an AOM in a phase-locked-loop configuration ensures an unlimited range for the channel stabilization, thus avoiding the need for a periodical reset of the actuator as in the case where a phase modulator was used, due to its finite phase adjustment range. The bandwidth of the phase-locked loop is 50 kHz, which is large enough to fully compensate the acoustic noise introduced by the fiber and the residual self-delayed laser noise and could be further extended with an optimized design of the electronics. Note that such a high control bandwidth can be achieved thanks to the use of classical beams for the sensing laser interference, which do not require photon counts integration procedures.

### The normalized interference pattern

The pattern produced by the classical interference of the QKD lasers in Charlie is modeled as $I=2{I}_{0}(1+\cos \varphi )$ where *I*_0_ is the lasers’ intensity, assumed equal, and *φ* their phase difference. In the experiment, we equalized the intensities of the two beams and aligned their polarization to maximize the contrast. We then considered the normalized interference pattern $\bar{I}=I/4{I}_{0}={\cos }^{2}(\varphi /2)$, which can be regarded as the classical counterpart of a single photon interference. Operating the interferometer in a condition where *φ* = 0 or *φ* = *π* corresponds to the case where all photons would be routed to one or the other port of the beamsplitter, i.e., operation in a dark port configuration. On the contrary, when the interferometer operates at *φ* = *π*/2 or *φ* = 3*π*/2, the probability of being detected on one or the other port are equal. In this condition, the phase fluctuations are directly mapped into intensity fluctuations, as in Fig. 3b. In our experiment, the residual phase fluctuations and its deviation are calculated from $\bar{I}$ inverting the related equation.

### Statistical methods

The variance of the phase ${\sigma }_{\varphi }^{2}$, or its corresponding deviation *σ*_*φ*_, at a given measurement time *t*_a_ can be directly calculated from time domain data, or as the integral of the power spectrum, which in turns is calculated from instantaneous phase data. In our experiment, we adopted both methods. First, we computed the Welch periodogram of the phase *S*_*φ*_(*f*), as retrieved from the interference pattern, and integrated it between the Fourier frequencies *f* = 1/*t*_a_ and *f* = *f*_s_/2, where *f*_s_ is the:

1 $${\sigma }_{\varphi }^{2}=\int\nolimits_{1/{t}_{{{{a}}}}}^{{f}_{{{{s}}}}/2}{S}_{\varphi }(f)\ {{{{{\rm{d}}}}}}f$$

We note that *f*_s_ must be at least twice as large as the noise bandwidth of the observed pattern to fulfill the Nyquist−Shannon sampling theorem^58^. In addition, we evaluated the phase variance over the time *t*_a_ by dividing the data set, composed of *N* phase samples *φ*_*j*_ and with total duration *T* = *N*/*f*_s_, in subsets of *n* points, where *n* ≈ *Nt*_a_/*T*. We then computed the variance of each subset and averaged over the number of subsets *i* ≈ *N*/*n*:

2 $${\sigma }_{\varphi }^{2}={\left\langle \frac{1}{n-1}{{{{{{{{\rm{{{\Sigma }}}}}}}}}}}_{j = 0}^{n}{({\varphi }_{j}-\bar{\varphi })}^{2}\right\rangle }_{i}$$

where $\bar{\varphi }$ is the average phase over each subset. We verified that both methods lead to the same result.

The obtained parameter is used to evaluate the QBER. When the interferometer is in the *φ* ≈ 0 condition and all the counts are expected to be on a single detector, the QBER represents the probability of having clicks on the complementary one. The contribution to the QBER from decoherence is hence calculated from the phase noise of the system according to the relation

3 $$e=\int \left(1-{\cos }^{2}(\varphi /2)\right)P(\varphi )\ {{{{{\rm{d}}}}}}\varphi =\int {\sin }^{2}(\varphi /2)P(\varphi )\ {{{{{\rm{d}}}}}}\varphi$$

As long as *φ* ≈ 0, which is the only interesting case in practice, it can be seen that Eq. (3) is simplified to $e={\sigma }_{\varphi }^{2}/4$, where ${\sigma }_{\varphi }^{2}$ is calculated from Eqs. (1) or (2). In Fig. 4 and throughout the text, we use this relation to evaluate the QBER.

### Single photon detectors

We employed a commercial fiber-coupled InGaAs/InP avalanche detector (Id Quantique ID230). The detector mounts a Stirling cooler that enables to cool down to −90 °C, reducing the dark counts related to the detection process to a negligible level. It operates in free-running mode, enabling asynchronous photon detection with 150 ps timing resolution, in a spectral bandwidth ranging from 900 to 1700 nm. The quantum efficiency is variable up to 25% and its dead time can be adjusted from 2 to 100 μs.

### Optical filtering

Our technique is based on the transmission in the same fiber of two separate signals, the QKD lasers, and the sensing laser, both in the C-band. Besides the issues related to nonlinear effects which generate background photons in the QKD lasers band, a key aspect is the efficient separation of the two signals in Charlie, to avoid that photons outside the QKD laser band reach the detector. This is primarily obtained with two cascaded 100 GHz-DWDM filters, each featuring 60 dB rejection at an offset of 1.5 nm from the central wavelength. However, the performances of standard telecom devices drop beyond 1300 nm, allowing a non-negligible power from the amplified spontaneous emission of the sensing laser, which extends to a wavelength of 1200 nm, to impinge the detectors. This was suppressed by placing a pair of additional free-space filters in front of the detectors, with nominal 50 dB rejection over the visible and near-IR band. Their 10 nm bandwidth, combined with the stronger selectivity of DWDM filters, ensured efficient filtering of the quantum photons. The overall losses of cascaded filtering stages amount to 2 dB, which is the result of the 84% transmissivity of the free-space filters and the coupling losses in the fiber/air interfaces.

## Supplementary information

Supplementary Information

## Data Availability

The processed data are available on Zenodo^59^.
