# Author Correction: Coherent phase transfer for real-world twin-field quantum key distribution

**DOI:** 10.1038/s41467-022-28447-w

**Published:** 2022-02-04

**Authors:** Cecilia Clivati, Alice Meda, Simone Donadello, Salvatore Virzì, Marco Genovese, Filippo Levi, Alberto Mura, Mirko Pittaluga, Zhiliang Yuan, Andrew J. Shields, Marco Lucamarini, Ivo Pietro Degiovanni, Davide Calonico

**Affiliations:** 1 INRIM, strada delle cacce 91, 10135 Torino, Italy; 2 INFN, sezione di Torino, via P. Giuria 1, 10125 Torino, Italy; 3 Toshiba Europe Ltd, 208 Science Park, Milton Rd, CB40GZ Cambridge, UK; 4 School of Electronic and Electrical Engineering, University of Leeds, LS29JT Leeds, UK; 5 Beijing Academy of Quantum Information Sciences, Building 3, West Area, No. 10 Xi-bei-wang East Road, Haidian District, 100193 Beijing, China; 6 Department of Physics and York Centre for Quantum Technologies, University of York, YO105DD York, UK

**Keywords:** Single photons and quantum effects, Quantum information, Fibre optics and optical communications, Optoelectronic devices and components, Optical metrology

Correction to: *Nature Communications* 10.1038/s41467-021-27808-1, published online 10 January 2022.

In this article, the funding from ‘the project EMPIR 19NRM06 METISQ - this project received funding from the EMPIR program co-financed by the Participating States and from the European Union Horizon 2020 research and innovation program.’ was omitted. The original article has been corrected.

