# Supplementary material for: Coherent phase transfer for real-world twin-field quantum key distribution
Source: Nat Commun. 2022 Jan 10;13:157. doi: 10.1038/s41467-021-27808-1 (PMC8748954; doi:10.1038/s41467-021-27808-1)
Supplement: Supplementary file 1 — Supplementary Information [file 41467_2021_27808_MOESM1_ESM.pdf]

Coherent phase transfer for real-world twin-field quantum key distribution
Supplementary Information

Cecilia Clivati[1,*], Alice Meda[1], Simone Donadello[1], Salvatore Virzì[1], Marco Genovese[1,2], Filippo Levi[1], Alberto Mura[1], Mirko Pittaluga[3,4], Zhiliang Yuan[5], Andrew J. Shields[3], Marco Lucamarini[6], Ivo Pietro Degiovanni[1,2], and Davide Calonico[1]

[1]INRIM, strada delle cacce 91, 10134 Torino, Italy
[2]INFN, sezione di Torino, via P. Giuria 1, 10125 Torino, Italy
[3]Toshiba Europe Ltd, 208 Science Park, Milton Rd, Cambridge CB40GZ, U.K.
[4]School of Electronic and Electrical Engineering, University of Leeds, LS29JT Leeds, U.K.
[5]Beijing Academy of Quantum Information Sciences, Building 3, West Area, No. 10 Xi-bei-wang East Road, Haidian District, Beijing 100193, China
[6]Department of Physics and York Centre for Quantum Technologies, University of York, YO105DD York, U.K.
[*]c.clivati@inrim.it

Supplementary Note 1: Frequency domain analysis of the phase fluctuations

As discussed in the main text, several noise processes affect the interference pattern at different timescales, which are better identified in a frequency-domain analysis. Supplementary Figure 1a shows the power spectral density of the phase fluctuations affecting the QKD lasers interference in an unstabilised (blue) and stabilised condition (red), as calculated from the interference traces shown in Figure 3 of the main Manuscript. In the unstabilised condition, a large portion of the noise is found in the lower part of the Fourier spectrum. The plateau observed between 10 Hz and 3 kHz is an artifact due to the limited range of the intererometer response, which wraps the phase into the $[0;\pi]$ interval. In practice, the phase noise rapidly diverges. This noise is considerably reduced

when the stabilization is activated.
The active noise cancellation is not effective at frequencies above 50 kHz. In particular, a significant noise contribution is observed at 0.9 MHz both in a stabilised and unstabilised condition, which corresponds to the servo-bump of the phase-locked loops between the QKD and reference lasers at the remote terminals. Above this frequency, the QKD laser noise becomes negligible as well. Between 30 kHz and 100 kHz, a characteristic pattern is observed in a stabilised condition, which is due to the self-delayed interference of the reference and sensing lasers. In presence of a length unbalance between the two arms ΔL, the lasers frequency noise, with power spectral density $S_{\nu,\mathrm{R}}(f)$ and $S_{\nu,\mathrm{S}}(f)$ is mapped into phase noise on the interference signal, with power spectral density

$$S_{\varphi}(f) = \frac{4}{f^2}\sin^2(\pi f n \frac{\Delta L}{c})[S_{\nu,\mathrm{R}}(f) + S_{\nu,\mathrm{S}}(f)] \quad (1)$$

where n is the refractive index of the fiber and c the speed of light. As can be seen, the noise contributed by the laser sources periodically nulls at Fourier frequencies $f = c/(n\Delta L)$, giving rise to the characteristic ripple observed in Supplementary Figure 1 [1]. In the lower part of the Fourier spectrum, the noise of the interference pattern shows the presence of a residual phase wander even in a stabilised condition. This is due to the combination of two distinct effects: the uncontrolled variations of optical paths which are not common between the sensing and the reference and QKD lasers, and the linear dependence of the optical phase on the laser wavelength. In fact, a change of the optical path δL_n generically depends on the changes of the refractive index δn and geometric length δL, and can be written as $\delta L_n = (\delta n L + n\delta L)$. This results in an accumulated phase $\delta\varphi_\mathrm{S} = 2\pi\delta L_n \nu_\mathrm{S}/c$ for the sensing laser, and $\delta\varphi_\mathrm{R} = 2\pi\delta L_n \nu_\mathrm{R}/c$ for the reference or QKD lasers. Because the optical length variations of the fiber are inferred from the sensing laser as $\delta L_n = \delta\varphi_\mathrm{S} c/(2\pi\nu_\mathrm{S})$, the QKD interference still suffers from uncompensated phase fluctuations whose power spectrum is

$$S_{\varphi,\mathrm{res}}(f) = S_L(f) \times (2\pi\frac{\nu_\mathrm{R} - \nu_\mathrm{S}}{c})^2 \quad (2)$$

where $S_L(f)$ is the power spectral density of the optical length variations δL_n. To quantify this term, we detected on two separate photodiodes the self-delayed interference of the sensing and reference lasers, obtained by beating each laser with itself after a round-trip from Charlie to the remote terminal and back. Here, we report only the results related to the Alice branch, where the environmental noise was found to be higher. We then sampled the phase of each beatnote separately and calculated the corresponding power spectrum $S_{\varphi,\mathrm{S}}(f)$ and $S_{\varphi,\mathrm{R}}(f)$, from which the spectrum of the optical length fluctuations of connecting fiber can be inferred as:

$$S_L(f) = \left(\frac{1}{2\pi}\frac{c}{\nu_\mathrm{S}}\right)^2 \times S_{\varphi,\mathrm{S}}(f) = \left(\frac{1}{2\pi}\frac{c}{\nu_\mathrm{R}}\right)^2 \times S_{\varphi,\mathrm{R}}(f). \quad (3)$$

From the above equations, the spectrum of the residual phase variations which are not common between the two can be calculated as

$$S_{\varphi,\mathrm{res}}(f) = \left(\frac{\nu_\mathrm{R} - \nu_\mathrm{S}}{\nu_\mathrm{S}}\right)^2 \times S_{\varphi,\mathrm{S}}(f). \tag{4}$$

The gray line in Supplementary Figure 1b represents $S_{\varphi,\mathrm{S}}(f)$ obtained from the measured phase of the self-delayed beatnote of the sensing laser after it has travelled a round-trip to Alice. The shadowed area shows the expected value of $S_{\varphi,\mathrm{res}}(f)$ as calculated from Eq. 4. The measured value for this quantity can be directly obtained by phase-comparing the self-heterodyned beatnotes at the two wavelengths in the RF domain. This measurement takes into account also other effects which have a different impact at the two wavelength, such as the length fluctuations of the short fiber paths which are not common between the two lasers, e.g. the DWDM couplers pigtails. The black line shows the measured power spectral density, which in fact overlaps with the expected value as calculated from Eq. 4 (shaded area). As a guidance, its trend is represented by the dashed black line in both Supplementary Figures 1a and 1b. Above 200 Hz the measurement is limited by the signal-to-noise ratio of the self-heterodyne beatnotes at detection. The good agreement with the measured noise on the QKD lasers interference (red trace in Supplementary Figure 1a) suggests that the wavelength difference is indeed the limiting factor, and that further increase in the long-term coherence can be expected by taking this into account in the design of the apparatus.
It is interesting to note as well that the gray curve in Supplementary Figure 1b represents the magnitude of phase fluctuations which would be observed in an unstabilised condition without the folding artifact as of Supplementary Figure 1a, and makes it evident that TF-QKD would not be possible in real networks without phase-stabilization.

Finally, we note that in presence of significant length unbalance between the two interferometer arms, once the residual fiber noise is reduced, the self-delayed noise of the reference and sensing lasers would emerge even if ultrastable, Hz-linewidth lasers are used. In our testbed, where the length mismatch is 44 km (considering both the service and QKD fibers), this noise contribution is less than one order of magnitude below the noise level we actually measured. However, by phase-locking the reference and sensing lasers using the optical comb, we ensured that the self-delayed laser noise of the two is highly correlated. Under this condition, because the self-delayed laser noise is detected together with the fiber noise by the sensing laser, we are able to correct for it on the QKD lasers interference as well.
In practice, mismatches up to tens of kilometers must be taken into account in realistic implementations, where the exact location of the terminals is bound by the network constraints. This would set an upper limit to the tolerated noise of independent laser sources. Notably, Eq. 1 allows a calculation of the interference phase noise contributed by the laser source as a function of length unbalance, once the lasers noise is measured experimentally. From it, the corresponding phase jitter and QBER can be computed following the procedure exposed in

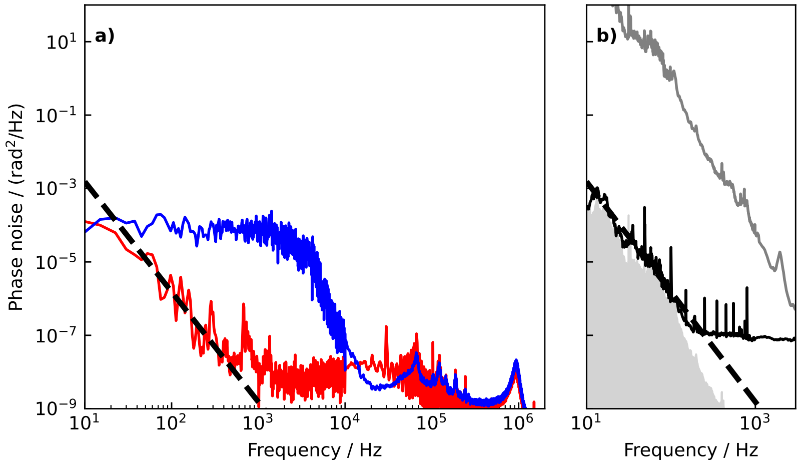

Supplementary Figure 1: **Phase noise of the QKD lasers interference and interpretation.** a) Phase noise power spectral density of the interference pattern between the QKD lasers in an unstabilized (blue) and stabilised condition (red). b) The phase noise measured by self-heterodyning the sensing laser in Charlie, after a round-trip to Alice (gray line). This was used to calculate the residual contribution expected on the interference pattern of QKD lasers (shadowed area). The black line shows the measured value for the differential noise at the two wavelengths, obtained by synchronously sending radiation at the two wavelengths in the link, and recording the beatnotes between the original and round-trip radiation in the two cases. The two beatnotes were then phase-compared in the RF domain. The dashed black line indicates the corresponding trend and is reported in (a) for guidance.

the main Manuscript (Methods section). This enables to estimate the maximum tolerated unbalance. Even if in principle the lasers frequency noise could be extrapolated from their linewidth or short-term instability, we stress that a direct measurement is desirable, as the corresponding jitter strongly depends on the specific spectral content, on the bandwidth over which they are stabilised and on the presence of spurs and other non-polynomial components.

Supplementary Note 2: Measurement of the interference with single-photon detectors

The measurement shown in Fig. 5 of the main Manuscript was obtained from the classical interference of QKD lasers on a fast photodiode, where we recorded the time-varying light-field intensity. We replicated the same measurement in photon-counting regime, attenuating the QKD lasers to $<100\,\mathrm{pW}$, and using a single-photon detector (SPD) in Charlie. In this regime, less than 10'000 photons/s reach the detector from each arm, making impossible a simultaneous measurement with a photodiode. To ensure that the same behaviour is observed with the two techniques, we performed the measurement in two stages: we first set the QKD lasers in Alice and Bob at their full power (2 mW of power launched in the fiber). In Charlie, we interfered them on a fiber coupler with two output ports, one of which was routed to a photodiode after amplification with an Erbium-doped fiber amplifier; the other was routed to the SPD after being attenuated by 80 dB. This allowed a direct comparison between the measured traces. In a second stage, we attenuated the QKD lasers in Alice and Bob, to resemble the operating conditions of a QKD experiment. Being impossible a comparison with an independent trace, we added a deterministic modulation on the optical phase and measured its signature on the interference pattern.
Supplementary Figure 2a shows the interference pattern simultaneously measured with classical beams (black) and with the SPD (red), when stabilization of the path was activated. In all measurements, the SPD was operated with an efficiency of 10% and an aperture time of 1 ms, and we subsequently integrated 25 adjacent samples. The photodiode measurements were averaged on the same timescale accordingly.
The visibility of the interference fringes measured on the photodiode and SPD traces were in agreement considering the uncertainty set by the Poissonian statistics in the quantistic measurement. To allow an easier comparison, the traces in the graphs have been normalised to the same scale. The overlap demonstrates the agreement between the results obtained with the two measurement techniques.
In Supplementary Figure 2b the same measurement is shown, in which a phase drift of $8\pi\,\mathrm{rad/s}$ was artificially inserted between the two beams. As expected, this generated periodical oscillations on the intensity and the detected number of photons at a 4 Hz rate. As in the previous case, the two measurements are

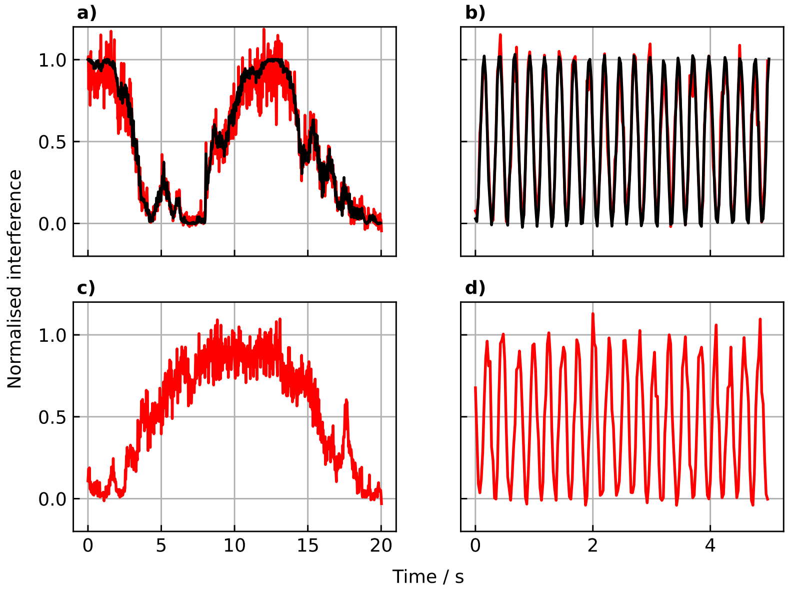

Supplementary Figure 2: **Observed interference patterns in classical and photon-counting regimes at various experimental conditions**. a) The interference pattern between classical signals recorded on a photodiode (black line) and in photon-counting regime, recorded with an SPD (red line), this latter obtained attenuating the interfering beams by 80 dB in front of the detector. b) Same as a, when a deterministic phase drift of $8\pi/s$ is inserted between the two. c) The interference pattern in photon-counting regime, when the QKD lasers were attenuated in Alice and Bob terminals. d) Same as c, when a deterministic phase drift of $8\pi/s$ is inserted between the two.

in agreement. This peculiar signature of the interference pattern was useful in the second stage of the measurement, in which the attenuation was placed in Alice and Bob. Supplementary Figure 2c and 2d show the results obtained with the SPD only, after attenuating the QKD lasers in Alice and Bob, when the phase was only subject to its residual wander after active stabilization (c), and when the artificial drift was inserted (d). Also in this configuration, the results reproduce the expected interference pattern.

Supplementary Note 3: Implementation of TF-QKD with phase stabilised optical paths

The setup described in the main Manuscript intentionally focuses on the stabilization of the fiber paths. The implementation of TF-QKD only requires a few additional components, namely the phase- and intensity-modulators that encode the quantum states and the encoding/decoding electronics. This part of the setup has been described extensively elsewhere both in laboratory-based and real-world setups, hence we will here focus on its integration inside the apparatus designed for the optical-path stabilization. Supplementary Figure 3 sketches a complete encoding apparatus for TF-QKD implementation in a phase-stabilised interferometer. Light from the local QKD laser at the Alice and Bob terminals is partly routed to a photodiode together with the incoming reference laser to enable the phase-lock, and partly sent to the encoding apparatus via polarization-maintaining fibers. Each encoding apparatus includes intensity and phase modulators (IM and PM respectively), a variable optical attenuator (VOA) that attenuates the beam to the single photon regime, and a beam splitter (BS) that spills out part of the radiation and sends it to a power meter for monitoring purpose. An additional IM must be included to produce the brighter photon fluxes required for the interferometer phase-stabilization when exploiting periodical realignment frames.
Suitable polarization control is adopted in Alice, Bob and Charlie to ensure a reliable phase-lock between the reference laser and local QKD lasers, and maximise the QKD interference signal upon recombination in Charlie. We note that the setup admits input and output ports only at the sensing laser wavelength, while the QKD radiation is generated locally. Still, the output port is in principle prone to trojan horse attacks. Access from outside can be prevented by inserting two or more optical isolators (>60 dB isolation each) and filtering stages which block radiation up to the edges of telecom bandwidth, where the performances of standard telecom components degrade. Even if there is no explicit study for trojan-horse against TF-QKD, the achievable isolation is more than enough to guarantee the security in standard BB84 run at 1 GHz [2]. In addition, suitable optical isolation can be inserted to prevent access from the phase-locked loop arm of the interferometer which might exploit non-negligible reflection at bulk components (modulators and photodiode).

Supplementary Note 4: Simulation of the expected key-rate and duty cycle

We estimated the achievable key-rate in a TF-QKD protocol implementation with active phase-noise cancellation following the protocol and notation proposed in [3] in the asymptotic scenario, with decoy states. Supplementary Table 1 reports the parameters used at input, while Supplementary Table 2 reports the simulation output. From Fig. 4 of the main Manuscript, we note that the

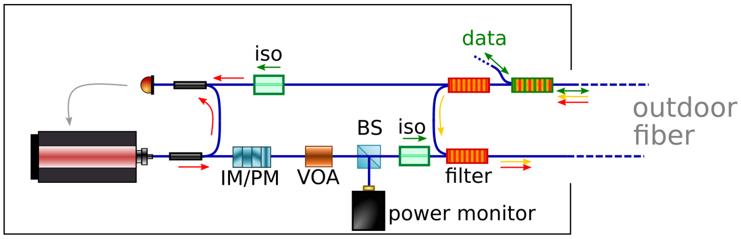

Supplementary Figure 3: Integration of TF-QKD components in a phase-stabilised interferometer. A sketch of the remote terminal where additional components are introduced to enable TF-QKD: iso: isolator(s), IM/PM intensity and phase modulator, VOA: variable optical attenuator, BS: beam splitter.

QBER contributed by the interferometer phase noise exceeds the 1% threshold in 0.4 s; conservatively, we can consider an upper bound of 1% within a 100-ms-long key transmission frame. The simulation parameters accounting for the phase noise have been set accordingly, while the others (e.g. f_{rep}, η_{c}, η_{det}, p_{dc}...) reproduce reasonable conditions, realisable in current experiments. Under these conditions, a secret key rate SKR of 4.942 kb/s is achievable, with a ratio over the PLOB bound of 10.83.

As detailed in the main Manuscript, two strategies are possible to suppress in real time the long-term wandering of the phase due to the differential fiber noise at the two wavelengths and guarantee a QBER lower than 1% for integration times longer than 100 ms: following the approach proposed in previous TF-QKD implementations, the quantum-states transmission can be periodically interleaved with high-intensity laser pulses carrying a reference phase and used to realign the interferometer phase [4]; or, owing to the higher phase-stability provided by our setup, an estimation of the residual phase can be alternatively derived on the basis of the measured QBER when the interferometer is set in the condition of Fig. 5 (lowermost panel) of the main Manuscript, where the relation between phase and QBER is linear [5]. An estimation of the achievable duty-cycle can be derived in both cases. From the values in Supplementary Table 1, the detection rate $D = 2u f_{\text{rep}} \eta_{\text{det}} \eta_{\text{c}} \sqrt{10^{-\alpha L/10}}$ amounts to 13 546 Hz, meaning that about 1355 counts are collected in a 100 ms time window. On the basis of previous experiments [5], we assume that 100 counts are sufficient to accumulate enough statistics and enable a good feedback when the latter strategy is chosen, the rest remaining available to generate the quantum key. The corresponding duty cycle (i.e. the ratio between reference unmodulated quantum pulses and normal quantum pulses) amounts to 92.6%, which is considerably higher than reported in all previous experiments. Instead, following the former approach, one can consider realignment frames with laser pulses 10-times brighter than the signal pulses, which brings a corresponding ten-fold improvement in D. In this case, only $\sim$0.7 ms are required to accumulate 100 counts and, accounting for another $\sim$0.7 ms to wait for the Double Rayleigh Backscattering to extin-

Symbol	value	Notes
L	206 km	Distance between Alice and Bob
f_{rep}	1 GHz	Alice's and Bob's source repetition rate
f_{EC}	1.1	error correction inefficiency
α	0.32 dB/km	fibre loss coefficient
n_{det}	2	number of detectors
p_{dc}	50 Hz	dark count probability per detector
η_{c}	0.75	Charlie's transmission
η_{det}	0.8	detector's efficiency
u	0.02 photons	photon flux decoy 1 and signal
v	0.2 photons	photon flux decoy 2
w	10^{-6} photon	photon flux decoy 3

Supplementary Table 1: List of parameters used to simulate the expected key-rate.

Symbol	value	Notes
D	13546 Hz	Detection rate
E_X	1.18%	QBER computational basis
$\bar{E}_Z$	12.46%	QBER phase basis
SKR	4942 bit/s	Secret key rate
SKR	4.942×10^{-6} bit/clock	Secret key rate
PLOB	4.562×10^{-7} bit/clock	Secret key capacity
SKR/PLOB	10.83	Ratio over PLOB bound

Supplementary Table 2: Quantities obtained from the simulation as a result of the calculation.

guish [4], one would achieve a 98.6% duty cycle. Considering that a part of the quantum signal must be used for the QBER evaluation, the effective duty cycle would not be lower than 90%.

Supplementary References

[1] Kefelian, F., Jiang, H., Lemonde, P. & Santarelli, G. Ultralow-frequency-noise stabilization of a laser by locking to an optical fiber-delay line. Opt. Lett. **34,** 914-916 (2009)

[2] Lucamarini, M. et al. Practical Security Bounds Against the Trojan-Horse Attack in Quantum Key Distribution. Phys. Rev. X **5**, 3 (2015).

[3] Curty, M., Azuma, K. & Lo, H.-K. Simple security proof of twin-field type quantum key distribution protocol. *npj Quantum Inf.* **5**, 64 (2019)

[4] Liu, H. et al. ArXiv:2101.00276v1 (2021)

[5] Minder, M. et al. Experimental quantum key distribution beyond the repeaterless secret key capacity. Nature Photon. **13**, 334-338 (2019).
